# Achievements and challenges in bioartificial kidney development

**DOI:** 10.1186/1755-1536-3-14

**Published:** 2010-08-10

**Authors:** Farah Tasnim, Rensheng Deng, Min Hu, Sean Liour, Yao Li, Ming Ni, Jackie Y Ying, Daniele Zink

**Affiliations:** 1 Institute of Bioengineering and Nanotechnology, 31 Biopolis Way, The Nanos, Singapore 138669, Singapore

## Abstract

Bioartificial kidneys (BAKs) combine a conventional hemofilter in series with a bioreactor unit containing renal epithelial cells. The epithelial cells derived from the renal tubule should provide transport, metabolic, endocrinologic and immunomodulatory functions. Currently, primary human renal proximal tubule cells are most relevant for clinical applications. However, the use of human primary cells is associated with many obstacles, and the development of alternatives and an unlimited cell source is one of the most urgent challenges. BAKs have been applied in Phase I/II and Phase II clinical trials for the treatment of critically ill patients with acute renal failure. Significant effects on cytokine concentrations and long-term survival were observed. A subsequent Phase IIb clinical trial was discontinued after an interim analysis, and these results showed that further intense research on BAK-based therapies for acute renal failure was required. Development of BAK-based therapies for the treatment of patients suffering from end-stage renal disease is even more challenging, and related problems and research approaches are discussed herein, along with the development of mobile, portable, wearable and implantable devices.

## Artificial kidneys

Treatment with an artificial kidney is the most widely applied therapy for kidney failure. Substantial improvements have been made in artificial kidney technology during the past decades, such as with regard to membrane technology, dialysate composition, and medication to address side effects. Despite these improvements, the high rates of mortality of critically ill patients with acute renal failure (ARF), ranging between 50% and 70%, did not change for several decades [1-5]. Also, the rates of morbidity and mortality of patients with end-stage renal disease (ESRD) receiving treatment with an artificial kidney remain high [6,7], and the survival advantage associated with renal transplantation is evident [8-12]. The problems associated with ESRD are increasing as the number of patients increases in industrialized countries, whereas the number of kidneys available for transplantation remains relatively low [6,7,13-15].

Which types of improvements of current therapies would be useful and which types of alternative therapies could be developed? It would definitely be useful to further improve artificial kidney technology to achieve a more efficient clearance of middle-sized uremic toxins such as β-2-microglobulin [16,17]. Also, prolonged or continuous and ambulatory modes of treatment of larger groups of patients would be desirable.

Continuous treatment with artificial kidneys is usually only performed in cases of ARF, in which patients are hospitalized. Concerning ESRD, only those patients with peritoneal dialysis receive prolonged or continuous treatment, which is a minority (< 10%, variable between different countries). The rest of the patients depend on traditional in-center hemodialysis, which is usually performed three times per week for several hours during daytime. This type of treatment not only greatly reduces the quality of life and is associated with high costs, but also leads to periodic accumulation of fluid, uremic toxins and metabolic wastes. Increasing evidence suggests that prolonged and/or more frequent therapies offer improvements in clinical outcomes and the quality of life, and might also be more cost-effective when performed at home [18-23]. Portable and wearable devices allow for a more normal lifestyle to be achieved and enable more frequent or continuous home-based therapies to be performed. A portable device for home hemodialysis that also allows for travel is now available [24-27]. A wearable artificial kidney is being developed, and successful human pilot studies have been performed [28-33]. This is currently one of the most exciting and promising developments in the field.

## Concepts and achievements in BAK development

Portable or wearable artificial kidneys would only allow for a certain extent of volume and solute control and removal of some uremic toxins. However, the kidneys have many additional functions. These include reabsorption of glucose, amino acids and water, and excretion of xenobiotics, drugs and other organic compounds [34-38]. Furthermore, the kidneys regulate the concentrations of sodium, potassium, phosphorus and divalent cations, and the acid-base balance [37,39,40]. They control blood volume and pressure, and have important metabolic and endocrinologic functions [37,41,42]. The hormones produced by the kidneys include erythropoietin, renin, prostaglandins and 1,25-dihydroxy vitamin D_3_, which is also called calcitriol and is the most active form of vitamin D. The kidneys are also the major source of the growth factor bone morphogenetic protein (BMP)-7 in the adult body, which appears to be important for bone homeostasis [43-45]. In addition, the kidneys may perform immunomodulatory functions [46-50].

As these complex functions cannot be provided by artificial kidneys, it was suggested that renal cells be included in the devices, and the concept of bioartificial kidneys (BAKs) was first developed by Aebischer and co-workers in 1987 [51-55]. BAKs based on this concept combined a conventional hemofilter, mimicking glomerular functions, in series with a bioreactor containing renal tubule-derived cells, which should provide tubular functions. Epithelial cells derived from the proximal tubules are most interesting for BAK development, because they perform a wide variety of functions, including reabsorption and secretion [34,35,37,38,40,41,56], as well as metabolic, endocrinologic [37,39,41,42,57] and probably also immunomodulatory [46-48,50] functions. The bioreactor unit seeded with proximal tubule-derived cells has also been called a renal tubule assist device (RAD) [58,59].

Research on BAKs has been performed mainly by two groups since the late 1990s: the group led by Akira Saito at the Tokai University School of Medicine, Kanagawa, Japan [60-71] and the group led by H. David Humes at the University of Michigan, USA [58,59,72-79]. Additional work on BAKs with a RAD-type bioreactor has been conducted by other groups [80,81], also with the goal to develop a device that clears from the blood toxins such as digoxin using cells overexpressing multidrug-resistant protein [82-84]. More recent work has addressed the development of a bioartificial glomerulus using CD133+ endothelial progenitor cells [85], and there was a conceptual study that proposed a bioartificial nephron-on-a-chip including glomerulus, proximal tubule and loop of Henle [86].

Clinical trials with BAKs have been performed by the group of H. David Humes and collaborators [76,78]. A Phase I/II clinical trial, which was performed with 10 critically ill patients with ARF, had shown that the device was sufficiently safe [78]. Any significant changes of parameters, which should be influenced by the human renal proximal tubule cells included in the device, could not be observed, and there were no significant changes in the pH of the ultrafiltrate or in 1,25-dihydroxy vitamin D_3 _levels. About 90% of the glutathione passed the RAD. There were some changes in the levels of the five cytokines tested. The levels of granulocyte colony-stimulating factor, interleukin-6 and interleukin-10 were significantly reduced in a subset of patients.

Subsequently, a multicenter, randomized, controlled, open-label Phase II clinical trial was performed in 2004 and 2005, which enrolled 58 critically ill patients with ARF [76]. 18 patients received continuous renal replacement therapy (CRRT), whereas 40 patients were treated using continuous venovenous hemofiltration (CVVH) and received additional treatment with a RAD. Patients were treated for up to 72 h. The results showed effects on 28-day and 180-day survival, which were improved in patients receiving CVVH plus RAD treatment. Only the effects on long-term survival (180 days) were significant.

This study was revolutionary but was also heavily criticized. It was pointed out that the study was severely underpowered [87]. Amongst the various points of criticism raised was also the issue that it was difficult to understand how long-term survival could be improved when no significant short-term effects were observed, in particular when the treatment lasted at most for only 72 hr [87]. A follow-up Phase IIb bridging study enrolling 53 patients was discontinued in 2006 after an interim analysis because it was not expected that the study would meet its efficacy goal (discussed in [76]).

The clinical trial was sponsored by RenaMed Biologics, Inc., a company co-founded by H. David Humes. The company was founded in 1995 as Nephros Therapeutics, Inc. and changed its name in 2005. After suspension of the Phase IIb bridging study in 2006, RenaMed Biologics, Inc. was restructured and renamed Nephrion, Inc. in 2007. Nephrion Inc. is now called CytoPherx, Inc., and it is engaged in the commercialization of a selective cytopheretic inhibitory device [88,89].

A first publication on this device [88] was based on the data from the Phase IIb clinical trial with BAKs, which had to be discontinued. Only the data from a control subgroup were evaluated for this study [88], and this control group received treatment with a sham non-cell-containing (SCD) cartridge. Twelve of the 24 patients in this control group received systemic heparin anticoagulation, and the remaining 12 patients received regional citrate anticoagulation. The results indicated improved survival in the citrate-treated group (67%, 90-day survival), whereas only 25% of the heparin-treated patients survived after 90 days. The results have to be interpreted with care owing to the small numbers of patients, but they were in accordance with other observations suggesting improved survival with citrate anticoagulation as compared to heparin treatment [90]. The authors of the study on the selective cytopheretic inhibitory device [88] interpreted their results in a way that lymphocyte attachment to the "cytopheretic" membranes in the cell-free cartridge (commercial F40 hemofiltration cartridge; Fresenius AG, Bad Homburg, Germany) improved clinical outcomes.

Altogether the work and the results discussed above suggested that there were significant challenges with the cell-containing cartridges and BAKs in clinical trials. It would be useful to discuss and review the work conducted so far and the device design presented in the interest of advancing new concepts for future research.

## Renal cell types and growth substrates applied in BAKs

What distinguishes a BAK from conventional hemofiltration devices is the bioreactor unit with renal cells. So far, BAK-related research has focused on renal proximal tubule-derived cells. Human primary renal proximal tubule cells (HPTCs) have been used in the clinical trials [76,78]. In contrast, most of the preceding experimental work and the animal studies performed by H. David Humes and co-workers have been performed with porcine primary renal proximal tubule cells [58,59,74,75] (the earlier studies [58,59] claimed that proximal tubule progenitor cells were used, although no evidence has been presented that a specific subfraction of progenitor cells had been isolated from the porcine kidneys). Also, the proximal tubule-like porcine cell line LLC-PK_1 _(Lewis lung cancer-porcine kidney 1) has been used frequently for BAK research [51,52,54,62-65,67], and part of the work has been performed with other animal-derived cell lines, which were not always of proximal tubule origin, such as Madin-Darby canine kidney (MDCK) cells.

**Figure 1:**
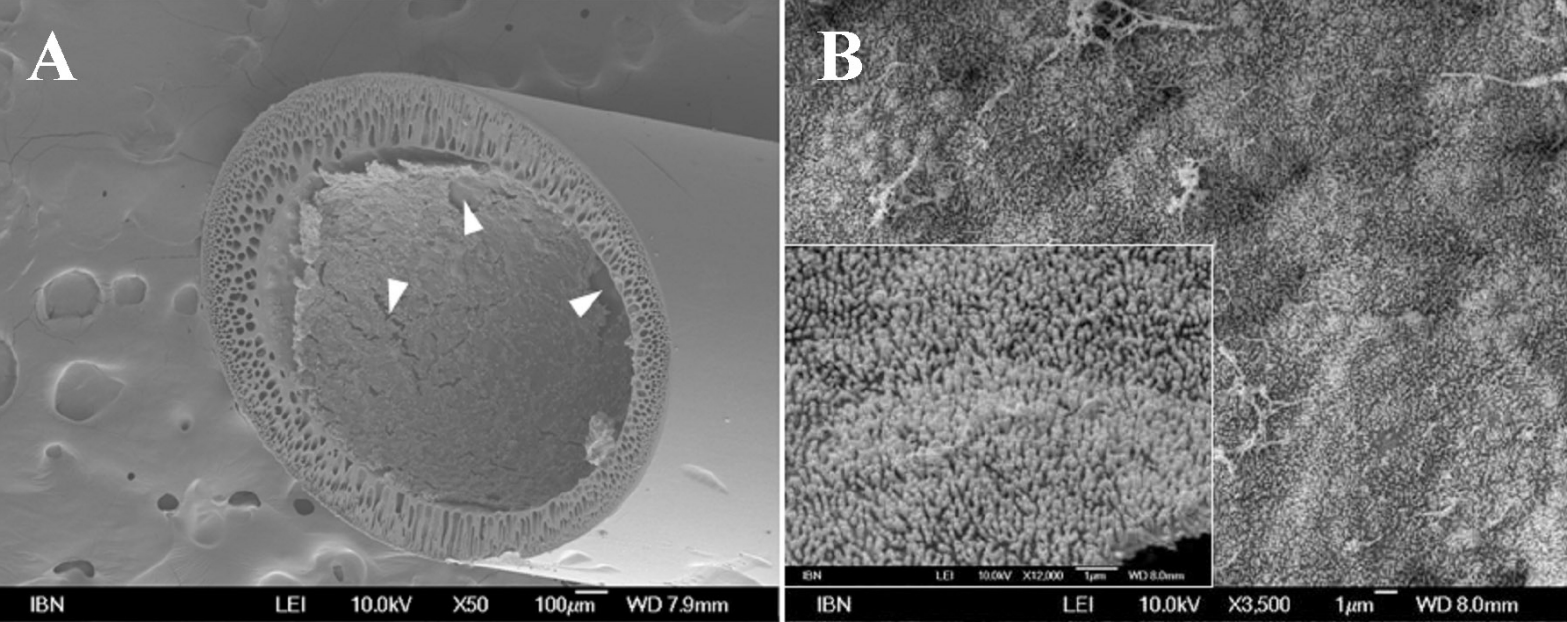
**Madin-Darby canine kidney (MDCK) cells form a polarized epithelium on the inner surface of polyethersulfone/polyvinylpyrrolidone (PES/PVP) hollow fiber membranes**. **(a) **Confluent monolayer of MDCK cells on the inner surface of a PES/PVP hollow fiber membrane. Cracks in the monolayer (indicated by arrowheads) are artifacts resulting from sample preparation (scale bar = 100 μm). **(b) **Luminal surface of the MDCK cell layer on the inner surface of a PES/PVP hollow fiber membrane (scale bar = 1 μm). The cell surface is densely covered with microvilli. The inset in the lower left corner shows an enlargement of the microvilli-covered surface (scale bar = 1 μm). All images were obtained by scanning electron microscopy.

It is mandatory that the renal cells form a confluent differentiated epithelium sealed by tight junctions on the porous membranes of the device. If this does not occur, the cellular functions would be absent or compromised. Under such conditions, the entire BAK would only perform the functions of a normal hemofiltration device, with the undesired diffusion of ultrafiltrate components back into the blood in the bioreactor unit. The problem with using animal cells for BAK research is that animal cell lines and probably also primary animal cells would show different requirements for growth and differentiation from the primary human cells. This is obvious, for instance, with regard to the formation of differentiated epithelia on different membrane materials. Figure 1 shows hollow fiber membranes consisting of polyethersulfone/polyvinylpyrrolidone (PES/PVP), within which MDCK cells form a polarized epithelium with a well-developed brush border. No such results could be obtained with HPTCs, which would not grow and survive on PES/PVP, regardless of whether it was coated with an extracellular matrix (ECM) or not (M. Ni, J. C. M. Teo, M. S. bin Ibrahim, K. Zhang, F. Tasnim, P.-Y. Chow, D. Zink and J. Y. Ying, unpublished results). It was also found that HPTCs would not grow well on polysulfone (PSF) membranes or membranes consisting of PSF blended with a phospholipid polymer; membranes coated with a fibronectin ECM did not improve the situation [68]. In contrast, MDCK and LLC-PK_1 _cells formed confluent monolayers on these materials, regardless of whether an ECM coating was present [68]. Furthermore, we found that HPTCs would not grow and survive on polysulfone/polyvinylpyrrolidone (PSF/PVP) membranes, and in this case, cell performance could not be sufficiently improved by a single coating of a suitable ECM consisting of collagen IV (M. Ni, J. C. M. Teo, M. S. bin Ibrahim, K. Zhang, F. Tasnim, P.-Y. Chow, D. Zink and J. Y. Ying, unpublished results).

Commercial cartridges with hollow fiber membranes consisting of PSF/PVP have been applied in BAKs for animal studies and clinical trials, and cells were seeded after ECM coating [58,74-78]. In many cases, ECM coatings of either laminin or collagen IV were used, and systematic tests revealed that these were indeed the most suitable ECM components for applications with HPTCs [91]. However, the results discussed above show that cell performance was mainly influenced by the underlying membrane material, and a single ECM coating would not sufficiently improve cell performance.

Together, the findings suggested that PSF/PVP was not suitable for applications with HPTCs, which was the cell type used in clinical trials. The fact that most of the *in vitro *and *in vivo *work on BAKs was not performed with HPTCs, while HPTCs were then applied in clinical trials, might explain some of the problems experienced. It would be important to perform future *in vitro *and preclinical studies with exactly the same cell type that would be used in clinical trials.

## Improvement of HPTC performance, alternative cell types and growth factor-releasing BAKs

An important question is whether HPTCs are indeed the most useful cell type for BAKs or whether other cell types might be more appropriate. HPTCs are obtained from nontransplantable human kidneys. Material from diseased kidneys would be suboptimal because, depending on the underlying condition, cell functions might be altered. Thus, it would be better to use only nondiseased kidneys that have been rejected for other reasons, although it might be difficult to obtain sufficient amounts of material. The limited cell source is indeed a serious problem, as the primary cells have a limited lifespan. As the membrane area of the bioreactor unit of one BAK should have a size of about 0.7-1.0 m^2 ^[70,76,78], it is questionable whether sufficient numbers of HPTCs can be obtained for the regular applications of BAKs and the commercialization of this approach.

Furthermore, primary proximal tubule cells show functional changes during passaging, and dedifferentiation as well as transdifferentiation processes occur [91-95]. This, together with the interdonor variability, makes standardization difficult; at the minimum, it would require extensive functional characterization of each cell batch at defined passage numbers.

**Figure 2:**
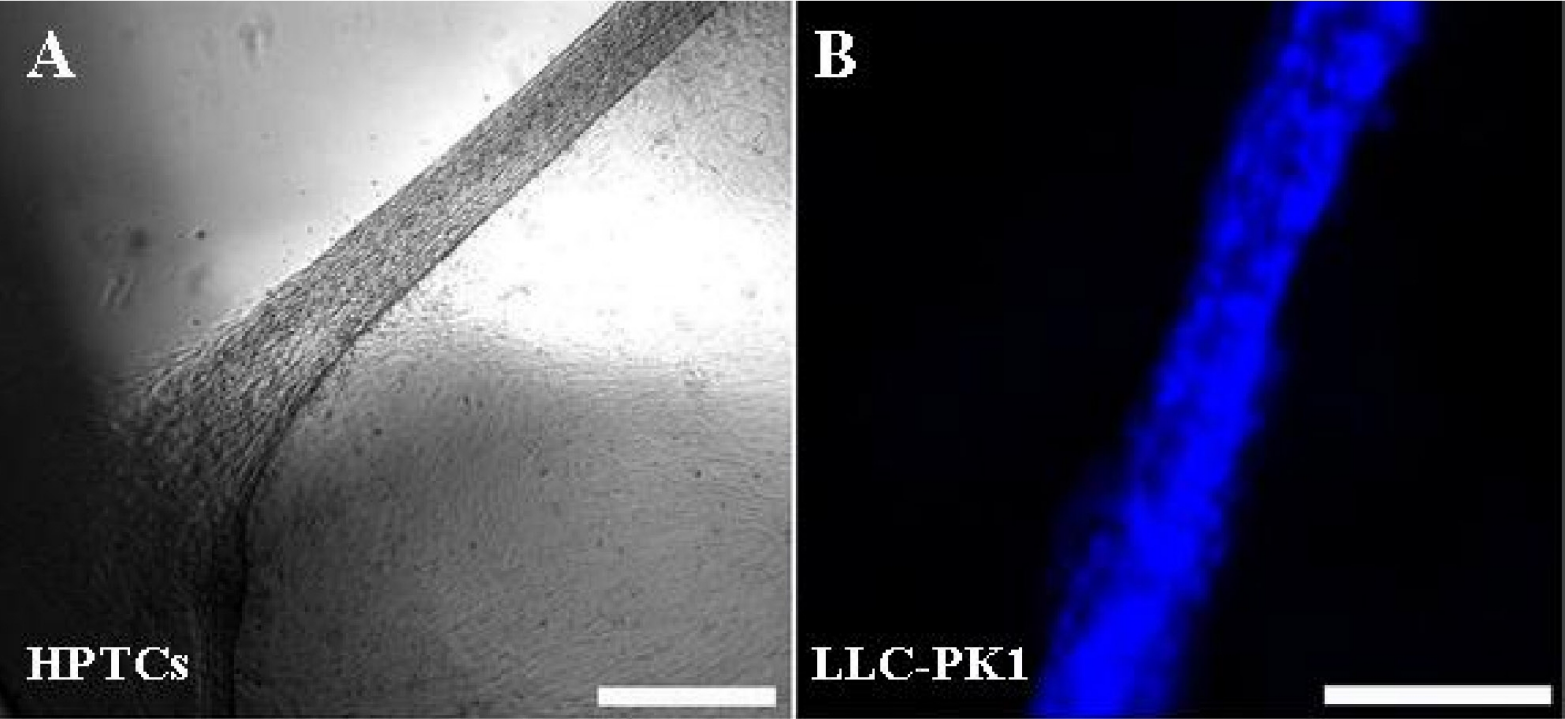
**Renal tubules that have formed spontaneously on 2-D surfaces *in vitro***. Renal cells were cultivated on the bottom of the wells of multiwell plates for several days until spontaneous tubule formation occurred. **(a) **Part of a renal tubule formed by human primary renal proximal tubule cells (HPTCs). The left end of the tubule is attached to the edge of the well (dark rim in the lower left corner). The epithelium on the bottom of the well was partially disrupted during the process of tubule formation (area devoid of cells in the upper left corner). The image was obtained by bright field microscopy, and the appearance of areas with bright and dark illumination is due to optical effects close to the edge of the well and around the tubule. **(b) **Part of a renal tubule formed by LLC-PK_1 _cells. The cell nuclei were stained with 4', 6'-diamidino-2'-phenylindole (DAPI, blue). The image was obtained by epifluorescence microscopy. Scale bars = (a) 400 μm and (b) 100 μm.

In addition, recent results showed that HPTCs form spontaneously large and functional kidney tubules on 2-D surfaces and within tubular substrates (Figure 2) [96]. The epithelium becomes disrupted during the process of tubule formation. Although such renal tubules generated *in vitro *in a gel-free system are very interesting for other applications, such as *in vitro *nephrotoxicology, their appearance in BAKs would compromise device functions and lead to clogging of the hollow fibers. Interestingly, Humes and Cieslinski described the formation of renal tubules from primary rabbit proximal tubule cells on 2-D surfaces in 1992 [97]. However, this phenomenon, which is in our experience a serious obstacle in BAK development, was not further addressed by Humes and Cieslinski. One reason for this could be the finding that tubule formation on 2-D surfaces was dependent on supplementation with different factors, including transforming growth factor (TGF)-β1 [97]. Thus, tubule formation would not occur spontaneously. However, we found that *in vitro *cultures of HPTCs expressed TGF-β1 and did not depend on supplementation; this was in agreement with our observation that formation of renal tubules by HPTCs occurred spontaneously [96].

Recent results showed that inhibition of tubule formation by HPTCs could be achieved by either co-culturing of HPTCs with primary human umbilical vein endothelial cells or by supplementation with 1 nM of BMP-7 (Tasnim et al., unpublished results). The latter finding was particularly interesting. BMP-7 (also called osteogenic protein (OP)-1) is a growth factor of the TGF-β superfamily and counteracts TGF-β1-induced effects [98-101]. The kidney is the major source of BMP-7 in the adult body, and BMP-7 appears to be important for bone homeostasis [43-45]. Animal experiments have shown that administration of BMP-7 improves kidney recovery in models of acute and chronic renal disease [44,100,102-107]. BMP-7 also has positive effects on vascular calcification and bone disease associated with chronic kidney failure in animal models [108-114]. Stryker Biotech is currently developing BMP-7-based treatments for kidney disease. It provides products consisting of a collagen sponge releasing human recombinant BMP-7 (OP-1 Putty and OP-1 Implant) for the treatment of bone disease.

**Figure 3:**
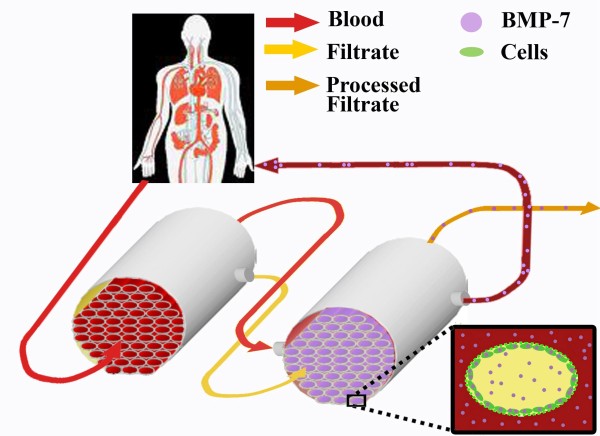
**Schematic of a bone morphogenic protein (BMP)-7-producing bioartificial kidney (BAK)**. The patient's blood (red) first enters the hemofiltration unit (left), which contains hollow fiber membranes for ultrafiltration. The blood and the ultrafiltrate (yellow) leaving the hemofiltration unit then flow into the bioreactor unit (right), which contains hollow fiber membranes with an epithelium of renal cells (green) on the inner surfaces. The cells secrete BMP-7 (violet), which becomes enriched in the ultrafiltrate during processing and in the blood flowing on the outside of the hollow fiber membranes. The blood enriched in BMP-7 flows back into the patient, and the processed ultrafiltrate (orange) is discarded. An enlarged cross-section of a hollow fiber membrane from the bioreactor unit is shown in the lower right corner. The ultrafiltrate flows in the lumen of the hollow fiber membrane, and the blood flows on the outside. The inner surface of the hollow fiber membrane is covered with BMP-7-secreting renal cells, and BMP-7 becomes enriched in the ultrafiltrate and the blood in the bioreactor unit. BMP-7 in the ultrafiltrate would regulate HPTC performance, whereas BMP-7 in the bloodstream would be delivered to the patient.

Treatment of kidney disease would require systemic administration of BMP-7, and in this regard, the short serum half-life of the growth factor of about 30 min is a problem. Treatment based on frequent administration of BMP-7 to kidney patients would be associated with high costs. A BMP-7-releasing BAK, which delivers low concentrations of the growth factor to kidney patients during extended time periods (Figure 3), might be an elegant solution for these problems. BMP-7 could either be released in a controlled manner from the membranes or other parts of the device, using, for instance, microparticle technology. Alternatively, HPTCs, which normally do not express BMP-7, could be genetically engineered to achieve BMP-7 secretion. A third possibility would be the inclusion of additional renal cell types (e.g., distal tubule cells) that express endogenous BMP-7. However, with the latter approach, it might be difficult to exactly control the amounts of BMP-7 released by the device. Apart from the beneficial effects on kidney patients treated with the BMP-7-releasing BAK, BMP-7 secretion within the device would also be expected to inhibit tubule formation and improve cell performance within the BAK. However, we would like to point out that the benefit of BMP-7 release by BAKs is still speculative at this point, and the discussion above reflects our personal view. It is also worth mentioning that secretion of active proteins into the bloodstream is one possible application of BAKs, and the use of BMP-7 is one possible example of such an application.

However, the question remains whether HPTCs, even if their performance can be improved, are indeed the best cell type for BAKs. Problems with the limited cell source, limited proliferative capacity, dedifferentiation, transdifferentiation and interdonor variability will remain. Also, for genetic engineering approaches, permanent cell lines would be preferable. For these reasons, Akira Saito and co-workers focused their work on cell lines, and the porcine cell line LLC-PK_1 _has been used in most of their studies. This cell line, which is frequently applied in *in vitro *nephrotoxicology, is very well characterized; as compared to other proximal tubule-derived cell lines, LLC-PK_1 _cells are relatively well differentiated [92]. However, the results of the work by Saito's group suggest that the passage of blood urea nitrogen (BUN) and creatinine when LLC-PK_1 _cells are used in a BAK is not appropriate for clinical applications [64], and the use of other cell types is suggested. Furthermore, multilayered overgrowth of LLC-PK_1 _cells in hollow fiber membranes was observed, which led to functional impairment after about 13 days [63]. Overgrowth could be inhibited with the mitogen-activated protein/extracellular signal-regulated kinase kinase inhibitor U0126 [65], but it was unclear what effects this inhibitor might have on kidney patients in BAK applications.

Recently, we have observed that LLC-PK_1 _cells also spontaneously formed renal tubules in gel-free cultures (Figure 2), and some of the multicellular structures observed by Saito's group [63,65] might have been generated by tubule-forming processes and might not just represent overgrowth. Apart from these specific problems with LLC-PK_1 _cells, animal-derived cells are generally problematic due to (1) the concerns associated with approval for clinical applications and (2) their different physiology. Therefore, it would be preferable to apply human cells instead of porcine cells in BAKs, but are appropriate human cell lines available?

The first permanent human proximal tubule-derived cell lines have been generated in the 1990 s by using oncogenes [115,116]. These cell lines include the widely used HK-2 cells. HK-2 cells show some differentiated functions of proximal tubule cells, but are functionally and morphologically not equivalent to primary cells [91,116-118], and the use of oncogenes is associated with safety concerns. Drawbacks due to high and uncontrolled levels of oncogene expression have been addressed by using a temperature-sensitive mutant of the simian virus (SV) 40 large T antigen [119], also in combination with the human telomerase reverse transcriptase (hTert) gene [120] for the conditional immortalization of HPTCs. Retroviral constructs were used in these studies, and the use of retroviral vectors, especially in combination with an oncogene, is associated with safety concerns in clinical applications. Also, the human proximal tubule-derived cell lines obtained did not express some important functional proteins, and further functional characterization would be required. In general, expression of important functional proteins or their mRNA is often markedly lower in renal cell lines than in primary cells, and this applies in particular to proximal tubule cells [121].

The SV40 T antigen has also been used in combination with hTert to generate reversibly immortalized HPTCs, taking advantage of the Cre/lox system [117]. The transgenes can be cut out when sufficient cell numbers have been obtained. The results showed that differentiated functions such as α-methylglucopyranoside uptake (indicator for glucose transport) or γ-glutamyl transferase (GGT) activity were compromised in comparison to primary HPTCs, and the levels of activity observed in primary cells were not restored after removal of the transgenes. This might be due to stable epigenetic changes.

Another approach to generate human proximal tubule-derived cell lines with improved properties for various applications is based on the expression of the hTert transgene only and does not involve oncogenes. The results suggested that expression of hTert alone was sufficient to achieve immortalization of HPTCs [118]. Characteristic features and functions of differentiated HPTCs did not appear to be severely compromised after immortalization. The hTert-expressing cells were obtained by using retroviral vectors, and this would be a safety concern in clinical applications. Also, the cells were not cloned after transduction, and thus the existing mixture of different immortalized cell clones might change over time. These issues should be addressed for applications in BAKs. It is worth mentioning that hTert-immortalized human proximal tubule-derived cells as well as the cell lines mentioned above are very interesting for a variety of other applications such as *in vitro *nephrotoxicology.

Stem cell-based approaches are most attractive for achieving an unlimited and less variable cell source for BAKs. It has been shown that murine embryonic stem (ES) cells cultured *in vitro *can be induced to express markers specific for the intermediate mesoderm, from which the kidneys arise during embryonic development, by using a combination of retinoic acid, activin-A, and BMP-7 [122]. The treated ES cells appear to be primed to respond to inductive signals and to differentiate along the renal epithelial lineage, although no true renal epithelial cells have been obtained *in vitro*. Another recent study demonstrated the induction of markers specific for renal precursors using similar inducers applied to *in vitro *cultivated human ES cells [123].

Wnt signaling is important for the proper development of kidney tubules in mice [124,125], and improper stimulation of the canonical Wnt pathway plays a role in various types of human cystic kidney diseases [126]. It would be expected that Wnt signaling would be essential for the differentiation of renal precursor cells into epithelial renal tubule cells *in vitro*. Human mesenchymal stem cells differentiate into mature kidney cells, including epithelial renal tubule cells, in an organ-specific environment in rodent embryos [127,128]. *In vitro *conditioned medium from injured proximal tubule cells induced epithelial differentiation of human adipose-derived adult mesenchymal stem cells [129]. Although the results suggest potential utility of stem cells in kidney bioengineering, further work is required to develop protocols for the differentiation of stem cells into mature human renal proximal tubule cells and other renal cell types *in vitro*. Currently, such cells are not available for applications in BAKs.

If stem cell-based approaches should be developed, the relevant legislation of those countries where the BAK is to be applied must be carefully considered. In this regard, the use of mesenchymal or other types of adult stem/progenitor cells (the presence of adult renal stem or progenitor cells is controversially discussed) is less problematic, as compared to the use of embryonic stem cells. Applications of induced pluripotent stem (iPS) cells might be most attractive, but the use of oncogenes and integrating viral vectors for reprogramming is associated with safety concerns. The recently developed iPS cells free of vectors and transgene sequences may provide a solution for this problem [130-134].

In conclusion, from those cell types currently available, HPTCs appear to be most appropriate for clinical applications. However, the use of HPTCs is associated with many problems, and it would be important to explore stem cell-based and other alternative approaches. Whatever cell type is selected in the future, it would be important to carefully examine exactly this cell type under the actual BAK conditions during the preclinical phase before advancing to clinical trials.

## Membranes

As discussed above, commercial hemodialysis/hemofiltration cartridges with ECM-coated hollow fiber membranes consisting of PSF/PVP have been applied in BAKs, but such materials do not appear to be appropriate for applications with HPTCs. The PVP component appears to contribute to the problems in HPTC growth and survival (M. Ni, J. C. M. Teo, M. S. bin Ibrahim, K. Zhang, F. Tasnim, P.-Y. Chow, D. Zink and J. Y. Ying, unpublished results). Membranes consisting of pure PSF are hydrophobic, which leads to difficulties in the adhesion of hydrophobic serum proteins. Thus, to prevent protein adhesion, all modern PSF- or PES-based membranes for hemodialysis/hemofiltration contain hydrophilic additives, which is in most cases PVP [135]. Although these components improve the antifouling properties, it is not too surprising that highly sensitive primary cells do not perform well on nonadhesive membrane surfaces.

Ueda et al. suggested in 2005 the use of asymmetric membranes with one hemocompatible and one cytocompatible surface [68]. These authors described a membrane consisting of PSF blended with a phospholipid polymer, which was asymmetrically distributed between the skin and the sponge layer of the membrane. In a static *in vitro *test, platelets could only slightly adhere to the sponge layer surface, which had a higher content of the phospholipid polymer. MDCK and LLC-PK_1 _cells formed confluent monolayers on the more adhesive skin layer [68]. However, this did not apply to HPTCs, and thus this type of membrane would not be appropriate for use with HPTCs. In general, it would be preferable to use the relatively rough sponge layer for cell growth and to expose the smooth skin layer to the blood.

Ueda et al.'s development of improved asymmetric membranes with a hemocompatible surface and a cell-compatible surface represented a major advance in the field. This could be achieved not only by generating asymmetric distributions of membrane components, but also by asymmetric coating of the surfaces using antifouling agents such as polyethylene glycol on the blood-exposed side and adhesive coatings on the cell-exposed side. The development of dual-layered membranes with each layer composed of a different material would be interesting. Dual-layered hollow fiber membranes have been developed for gas separation and water purification [136,137]. Membrane materials that are currently employed in hemodialysis/hemofiltration with demonstrated hemocompatibility would be suitable for the blood-exposed layer. In addition, the surface of this blood-exposed layer could be conjugated to anticoagulants (e.g., heparin) to reduce the requirement for other conventional anticoagulation treatments.

The remaining challenge involves the selection of materials and coatings appropriate for the cell-exposed layer. Our recent findings revealed problems with HPTC survival and differentiation on a variety of commercially available membrane materials (M. Ni, J. C. M. Teo, M. S. bin Ibrahim, K. Zhang, F. Tasnim, P.-Y. Chow, D. Zink and J. Y. Ying, unpublished results). Various surface treatments and single ECM coatings did not lead to sufficient improvements. In contrast, after double coating of PES/PVP or PSF/PVP with 3,4-dihydroxy-L-phenylalanine (DOPA) and collagen IV, improvement of HPTC performance was observed and the cells formed confluent epithelia with tight junctions on the double-coated PSF/PVP.

As PVP appeared to be problematic, other additives were tested. HPTCs formed confluent epithelia on membranes consisting of PSF blended with FullCure (FC) under bioreactor conditions. Single or double coating did not further improve cell performance on such PSF-FC membranes (Ni et al., unpublished results). The fact that PCF-FC membranes do not require any coating for applications with HPTCs makes this material very attractive.

Growth and differentiation of primary human cortical tubular epithelial cells were also observed on collagen IV-coated thin film and nanostructured materials [138]. The materials tested included silicon nanopore membranes. These membranes have monodisperse slit-shaped nanopores and display greater selectivity at a given value of hydraulic permeability in comparison to conventional membranes with cylindrical polydispersed pores [138,139]. High hydraulic permeability would be particularly important for the development of miniaturized wearable or implantable devices.

## Challenges related to the development of portable, wearable and implantable devices

Current BAKs are large and immobile. The development of portable, wearable or implantable devices would be highly desirable and has been suggested by different authors during the past 20 years [53,62,70,140,141]. Such devices would allow for prolonged or continuous treatment, which would be expected to have beneficial effects on the patients' health status. In addition, higher mobility would substantially improve the patients' quality of life. Also, given the high costs associated with in-center treatment of ESRD, the costs associated with in-center BAK therapy with immobile devices might be a serious obstacle.

Portable artificial kidneys are already available [24-27], and clinical trials with wearable artificial kidneys are currently underway [28-33]. Thus, the engineering problems associated with miniaturization of cell-free artificial kidneys seem to be challenging but solvable. A problem specifically related to BAKs is the viability and functional performance of the cell layer. Currently, it is unclear whether renal epithelial cells, which react highly sensitively *in vitro *to the environmental conditions [142-144], can be maintained in a viable and functional state when they are moved around in a mobile device. Mobility will be associated with mechanical stress, which easily damages renal epithelial cells [142-144]. If the cells are functionally compromised, their reabsorption rate will decline. Removal of an excessive volume of extracellular fluid in the hemofiltration unit without sufficient reabsorption in the bioreactor will lead rapidly to a critical condition. Furthermore, if the cell layer in the device should become leaky, uremic toxins from the ultrafiltrate will diffuse back into the bloodstream. Thus, in a mobile device, the cell cartridge must be embedded in a way that damage by mechanical stress and other environmental factors is minimized, and a miniaturized system for monitoring the cell functions and the integrity of the epithelium would be required. Such housing and monitoring systems have yet to be developed, and it is unclear how cells could be shielded from mechanical stress.

Another challenge is the development of new membrane and device materials with improved anticoagulation and antifouling properties. Currently, the lifetime of hemodialysis/hemofiltration membranes is only about 100 hr. Exchange of cartridges would be relatively easy when an extracorporeal device would be used. However, an extracorporeal circulation is associated with a high risk of infection, and on-site handling requires extremely well-trained and dexterous patients. Given these and other problems, an implantable device appears to be preferable. However, even if the lifetime of the membranes could be dramatically extended to several weeks or months, this would still mean frequent surgery for an implantable device. Even for a device with a housing and an exchangeable cassette that would be implanted close to the body surface [141], the situation might not be acceptable.

Apart from the unsolved problems with the lifetime of membranes, it is currently not clear for how long an intact epithelium can be maintained in the bioreactor unit. Differentiated epithelia formed by HPTCs can be maintained under optimized *in vitro *conditions in the laboratory for several weeks. The lifetime of the epithelium might be shorter under BAK conditions whereby not all of the parameters can be optimized for cell performance. Thus, it is expected that the cell cartridge would also require frequent exchange, and this would apply to mobile as well as to immobile devices. This raises again the issue of cell source, as it would be difficult to obtain sufficient number of primary cells from nontransplantable organs. Thus, as long as the cell sourcing problem is not resolved, regular long-term treatment of a large number of ESRD patients would not be feasible with immobile or mobile devices.

## Conclusions

After 23 years of BAK research, there remain many challenges to be addressed. Given the problem of cell sourcing, the costs associated with in-center BAK treatment and the issues associated with mobile devices, the development of BAK-based therapies for ESRD patients would take many more years of intense research. The most straightforward path ahead may involve the development of immobile devices for the treatment of ARF. This was the approach followed by H. David Humes and co-workers, and the existing complications demonstrate that a great deal of further research would still be needed. In the future, it would be important to perform the experimental work with exactly the same cell type that would be used in clinical studies. Otherwise, it would be difficult to predict how the clinically relevant cell type would perform under the actual BAK conditions. Although currently HPTCs appear to be the most relevant cell type, primary cells are suboptimal, and this issue as well as the cell sourcing problem must be addressed. Also, it would be critical to design and synthesize novel membrane materials with cytocompatibility, antifouling and anticoagulation properties, as well as other features such as hydrodynamic permeability and selectivity. Progress in this multidisciplinary research area would provide a solid basis for the development of more advanced BAK-based therapies.

## Abbreviations

ARF: acute renal failure; BAK: bioartificial kidney; BMP-7: bone morphogenetic protein-7; BUN: blood urea nitrogen; CVVH: continuous venovenous hemofiltration; CRRT: continuous renal replacement therapy; DAPI: 4', 6'-diamidino-2'-phenylindole; DOPA: 3,4-dihydroxy-L-phenylalanine; ECM: extracellular matrix; ES: embryonic stem; ESRD: end-stage renal disease; FC: FullCure; GGT: γ-glutamyl transferase; HPTC: primary human renal proximal tubule cell; hTert: human telomerase reverse transcriptase; iPS: induced pluripotent stem; LLC-PK_1_: Lewis lung cancer-porcine kidney 1; MDCK: Madin-Darby canine kidney; OP-1: osteogenic protein-1; PES: polyethersulfone; PSF: polysulfone; PVP: polyvinylpyrrolidone; RAD: renal tubule assist device; SCD: sham non-cell containing; SV40: simian virus 40; TGF: transforming growth factor.

## Competing interests

The authors declare that they have no competing interests.

## Authors' contributions

DZ drafted the manuscript, and the other authors critically revised it. RD, MH, FT and YL contributed the figures. All authors read and approved the final manuscript.

## Acknowledgements

We thank Joscha Muck (Institute of Bioengineering and Nanotechnology (IBN), Singapore) for help with arranging the figures, and Kangyi Zhang (IBN, Singapore) and Mohammed Shahrudin bin Ibrahim (IBN, Singapore) for comments and discussions. This work is supported by the Institute of Bioengineering and Nanotechnology (Biomedical Research Council, Agency for Science, Technology and Research, Singapore).
